# Effect of intestinal microecology on postnatal weight gain in very preterm infants in intensive care units

**DOI:** 10.1186/s13099-021-00445-1

**Published:** 2021-08-02

**Authors:** Ying-Xue Ding, Shou-Ni Wang, Hong Cui, Li-Na Jiang

**Affiliations:** 1 Department of Pediatrics, Beijing Friendship Hospital, Capital Medical University, Beijing, 100050 China; 2 Department of E.N.T, Yantai Shan Hospital, Yantai, 264001 Shandong China

**Keywords:** Very preterm infants, Extrauterine growth, Neonatal intensive care unit, Intestinal microflora

## Abstract

**Objective:**

To study the effect of intestinal microecology on postnatal weight gain of very preterm infants in neonatal intensive care unit (NICU).

**Methods:**

Very preterm infants who met the inclusion criteria were enrolled. The subjects were divided into the extrauterine growth retardation (EUGR) group(defined as a body weight less than the 10th percentile of the corresponding gestational age or a weight loss between birth and a given time of  >  2SD were considered EUGR) and normal growth group, and the growth was evaluated at 2 and 4 weeks after birth. Meanwhile, the stool samples were taken to perform16S ribosomal RNA (rRNA) high -throughput 16S rRNA sequencing of the intestinal microflora was performed on stool samples.

**Results:**

A total of 22 infants were included. There was no significant difference in the alpha diversity indexes indices between the two groups at 2 weeks or 4 weeks after birth. The beta diversity analysis showed that the two groups had similar principal components of the intestinal microflora were similar between the two groups. Linear discriminant analysis (LDA) effect size (LEfSe) showed that 2 weeks after birth, the bacteria with an absolute LDA score (log10) higher than 4 included Streptococcaceae, Streptococcus, Bacteroidetes, Bacteroidales and Stenotrophomonas in the EUGR group and Enterococcaceae and Enterococcus in the control group. At the 4th week after birth, the bacteria with an absolute LDA score (log10) higher than 3 in the EUGR group includedwere Clostriaceae, Eubacteriaceae and Eubacterium. TheBy comparing the composition of the microbial community composition comparison showed, significant differences were found in the principal components of Enterococcus and Streptococcus on the family and genus levels at 2 weeks after birth. No Bifidobacterium was found in either group at 4 weeks after birth.

**Conclusion:**

Intestinal microecology is different between infants with EUGR and those with normal growth. The diversity and richness of the intestinal microflora in preterm infants at the NICU are significantly insufficient and change dynamically with time, and the establishment of intestinal homeostasis is obviously delayed.

**Supplementary Information:**

The online version contains supplementary material available at 10.1186/s13099-021-00445-1.

## Introduction

Preterm infants can suffer from immature immune system and physiological characteristics. The establishment of the neonatal intensive care units (NICU) and the extensive development in advanced life support for preterm infants have generally improved their survival rate. Very premature infants need to be admitted to the NICU after birth and receive respiratory, nutritional, antibiotic and other support treatment. In 2019, the incidence of preterm infants was reported by the World Health Organization (WHO) to be about 10.6%, with an average of 14.8 million premature births per year. Among these births, more than 1.1 million happen in China, with an incidence of 6.9% [1]. A common phenomenon is that these infants have different degrees of feeding intolerance and growth retardation, and it is challenging for them to establish a normal intestinal flora [2]. Healthy intestinal microecology plays an indispensable role in the neonatal intestinal development, maintaining the integrity of the intestinal mucosa and nutritional status of the host [3]. Therefore, maintaining the colonization of the normal intestinal microflora is undoubtedly a key factor contributing to the overall health of newborns. Extrauterine growth retardation (EUGR), considered as a body weight less than the 10th percentile of the corresponding gestational age or a weight loss between birth and a given time of  >  2SD, does not only affect the growth of infants, but also affects the occurrence and development of diseases, leading to prolonged hospitalization period and long-term development retardation, finally affecting the brain development and the occurrence of metabolic diseases in adulthood. In this work, we monitored very preterm infants with a gestational age of less than 32 weeks at the NICU of our hospital and investigated the association between growth and the intestinal microecology in order to find a new way to improve the extrauterine growth of very preterm infants.

## Materials and methods

### Study design

The subjects of this study included very preterm infants with a gestational age of less than 32 weeks who were admitted to the NICU of Beijing Friendship Hospital affiliated to the Capital Medical University from January to December 2018. A total of 22 very preterm infants who met the inclusion criteria were finally included. Physical growth was assessed at 2 time points, 2 weeks and 4 weeks after birth. The infants were divided according to the body weight into the EUGR group and normal growth group. Meanwhile, stool samples were taken and stored at – 80 ℃ to perform 16S ribosomal RNA (rRNA) high-throughput sequencing of the intestinal microflora (BeiJing Allwegene Technology Co. Ltd., 502, Building 3, Block C, Changyuan Tiandi, Suzhou Street, Haidian District, Beijing). Then, we analyzed the association between the extrauterine growth of very preterm infants and the intestinal microecology. This study was approved by the Ethics Committee of Beijing Friendship Hospital affiliated to the Capital Medical University. The infants' parents (or responsible relatives) gave written informed consent. The same nutrition strategy was followed with all the infants: all preterm infants were weaned within 24 h after admission. Early micro-feeding (10–15 ml/kg/d) was applied, if tolerable, then the amount of milk was gradually increased at the speed of 15–20 ml/kg/d. Parenteral nutrition support was given when total enteral feeding was not achieved.

### Physical growth evaluation

Physical growth was evaluated according to the Fenton growth chart for preterm infants (2013) [4] as follows: the infants with a body weight less than the 10th percentile of the corresponding gestational age or a weight loss between birth and a given time of  >  2SD were considered to have extrauterine growth retardation, and those between the 10th and 90th percentiles were considered to have normal growth.

### Inclusion and exclusion criteria

The inclusion criteria were as follows: (1) admission to the NICU immediately after birth; (2) a gestational age of 28–32 weeks, single pregnancy; (3) hospitalization time  >  28 days; (4) antibiotic treatment (Amoxicillin-potassium clavulanate/Piperacillin-tazobactam) after birth and for less than 5 days.

The exclusion criteria were as follows: (1) severe congenital malformations and congenital genetic metabolic diseases; (2) discharged automatically with unclear outcomes after discharge; (3) incomplete data; (4) received probiotics during the collection of stool samples; (5) mixed feeding.

### Stool sample collection

The collection of the stool samples of preterm infants strictly followed the principle of aseptic operation and was performed on the 14th and 28th day after birth using a disposable sterile stool container and preserved at – 80 ℃. Next, the samples were sent to Allwegene Technology Inc. to perform DNA extraction, sequencing and bioinformatic analysis.

### Bioinformatic analysis

The FLASH v1.2.11 software (Fast Length Adjustment of Short Reads, http://ccb.jhu.edu/software/FLASH/index.shtml) [5] was used to merge the sequencing data, and the chimeras were filtered using the VSearch v2.7.1 (https://github.com/torognes/vsearch) [6] software. Sequences with a similarity greater than 97% were defined as one operational taxonomic unit (OTU), and the representative sequences were accordingly selected. BLAST (https://blast.ncbi.nlm.nih.gov/Blast.cgi) was used for sequence alignment, and the QIIME v1.8.0 software (quantitative insights into microbial ecology, http://qiime.org/) [7] was used to analyze the α diversity, which represents the species richness in the sample, along with the composition of the samples, on the phylum, class, order, family and genus levels.

### Statistical methods

The SPSS 20.0 software was used for data analysis. First, we performed statistical matching grouping to remove the effects of the feeding mode, delivery mode and antibiotics, and only the effects of intestinal microecology on growth were analyzed. Count data, such as the gestational age (stratification), gender, delivery mode and feeding mode, were compared using the Pearson’s χ^2^ test. When the sample size was too small, the Fisher exact probability method was used for intergroup comparison. A p value  <  0.05 was considered to be statistically significant.

## Results

### Basic information of the subjects

Among the 22 very preterm infants included in the study, there were 14 males and 8 females. There were 8 cases at 28 to less than 30 weeks of gestational age, 6 cases at 30 to less than 31 weeks of gestational age and 8 cases at 31 to less than 32 weeks of gestational age. No significant differences were found in the gender ratio, delivery mode, feeding mode or gestational age stratification between the EUGR and control groups (Table 1).

**Table 1:** Clinical characteristics of the subjects

Group	Gender		Delivery		Feeding		Gestation age		
	Boy	Girl	Vaginal	Caesarean	Breast milk	Formular	28 w ≤ GA < 30 w	30 w ≤ GA < 31 w	31 w ≤ GA < 32 w
2 w									
Control	10	4	8	2	8	6	6	5	3
EUGR	4	4	6	6	4	4	2	1	5
* p* value	0.386		0.204		1.000		0.649		
4 w									
Control	9	3	7	5	6	6	5	4	3
EUGR	4	6	4	6	6	4	4	2	4
* p* value	0.092		0.392		0.691		1.000		

### Sequencing results and species accumulation curve

**Fig. 1:**
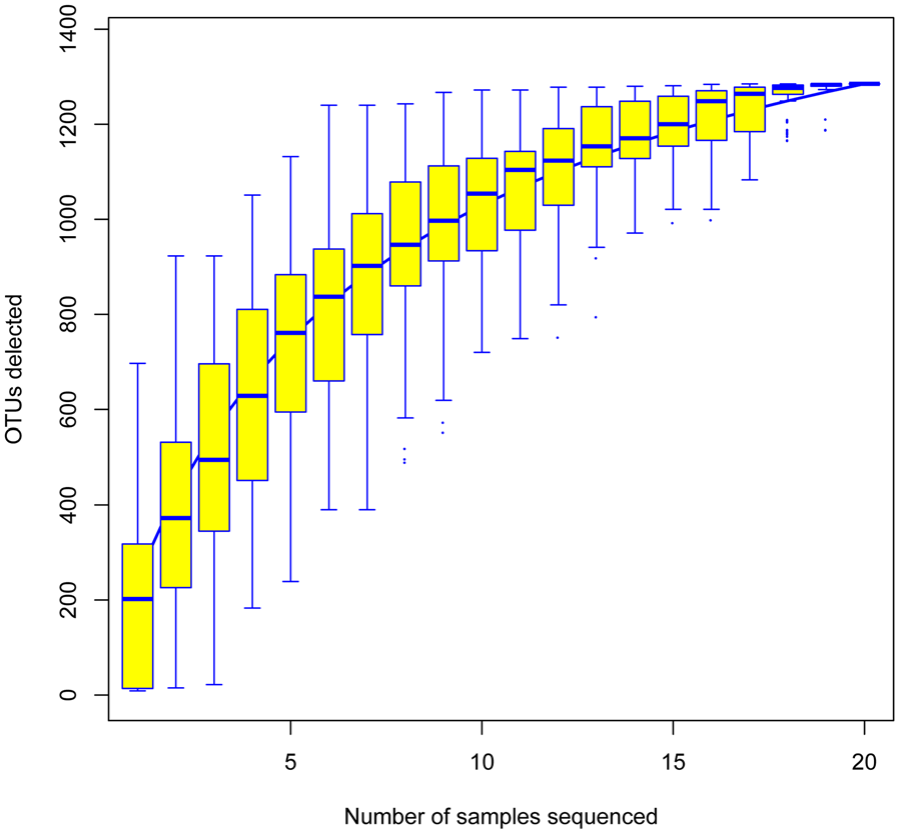
Species accumulation curve

DNA extraction and PCR were successfully performed on all the stool samples, and the MiSeq libraries were prepared and sequenced. The species accumulation curve showed that the number of OTUs rapidly increased as the stool samples increased at the beginning, then slowly increased and finally entered a stationary phase, which proved that the number of samples in this study was sufficient to reflect the species richness in the community (Fig. 1).

### OTU analysis

**Fig. 2:**
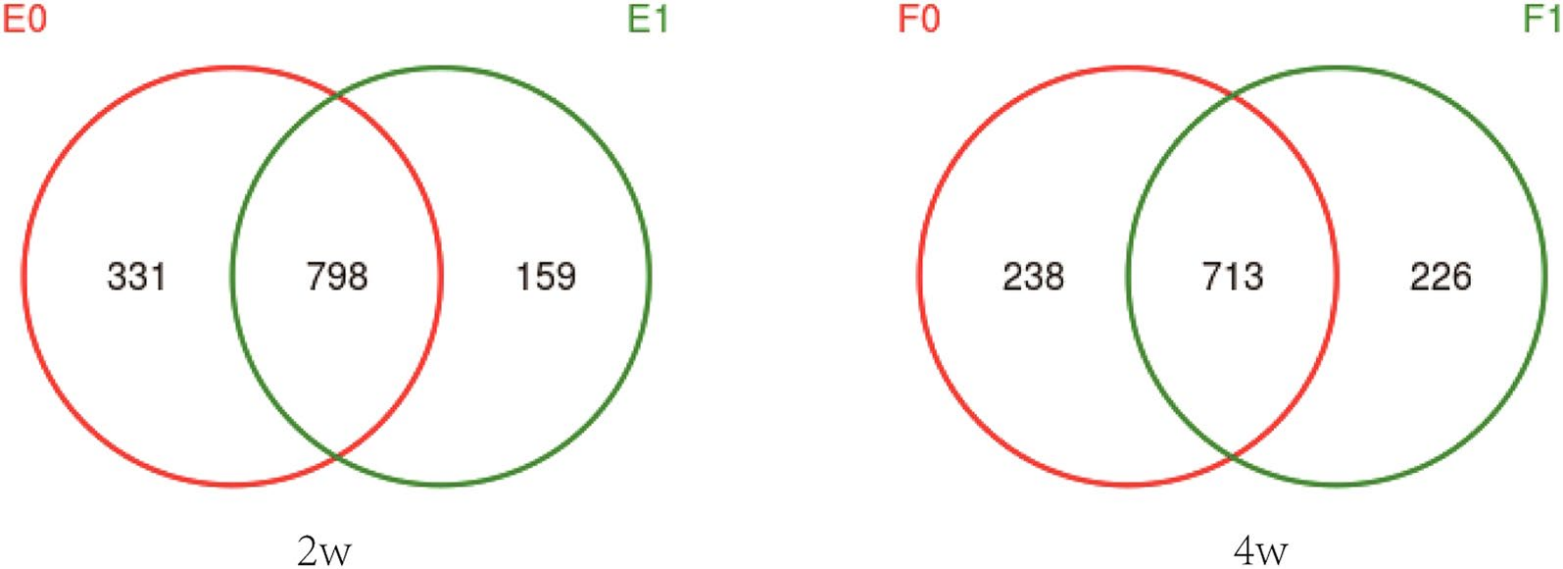
Venn plot. E0: control group, 2 weeks after birth; E1: EUGR group, 2 weeks after birth; F0: control group, 4 weeks after birth; F1: EUGR group, 4 weeks after birth. The overlapping areas of the circles with different colors indicate the shared OTUs, while the non-overlapping areas are OTUs specific to one group

On the 14th day after birth, 8 infants had growth retardation, and a total of 1288 OTUs were obtained, among which 331 were specific to the control group, 159 were specific to the EUGR group and 798 were shared by the two groups. On the 28th day after birth, 10 infants developed growth retardation, and a total of 1177 OTUs were obtained, among which 238 were specific to the control group, 226 were specific to the EUGR group and 713 were shared by the two groups (Fig. 2).

### Alpha diversity analysis

**Fig. 3:**
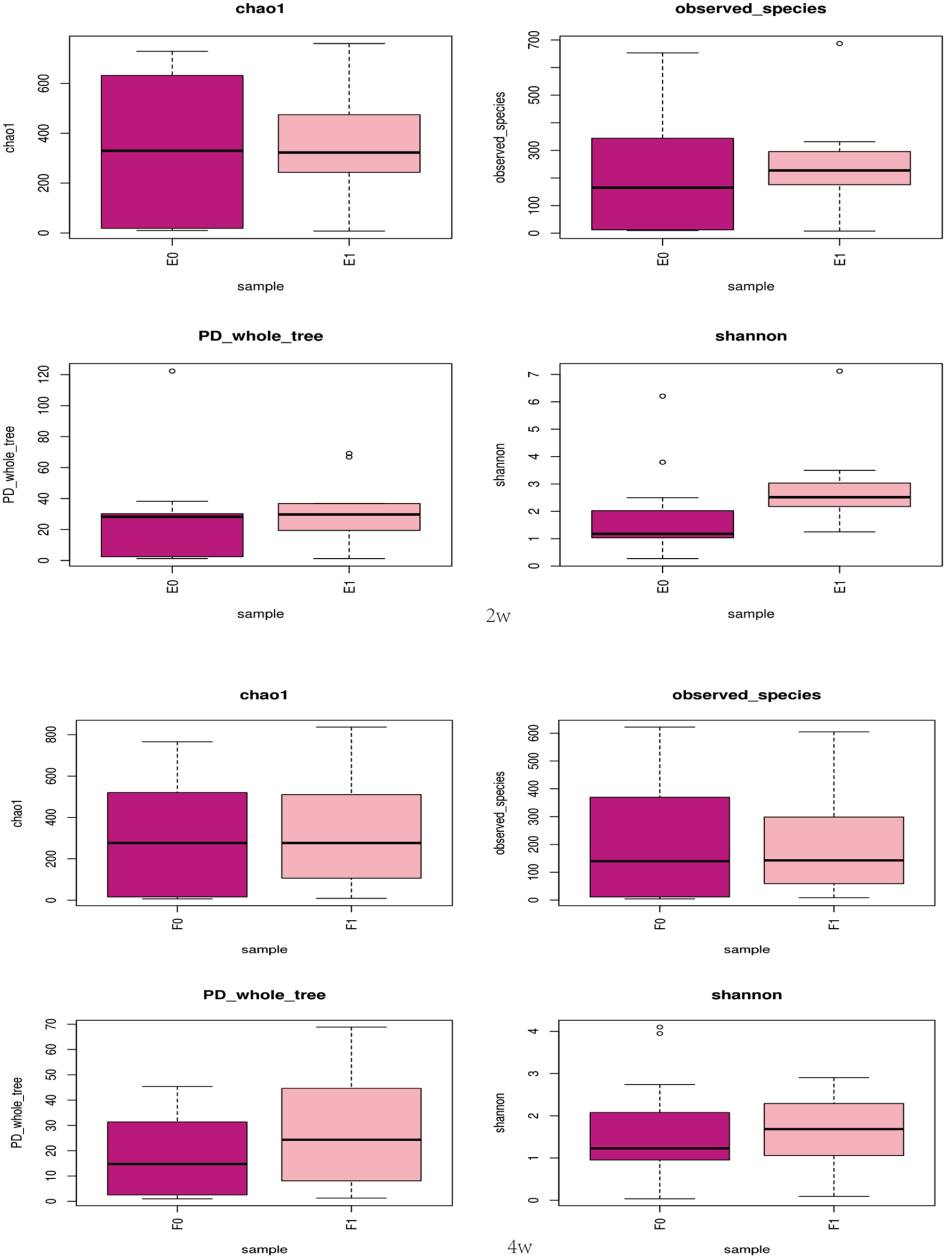
Alpha diversity analysis of the two groups at different postnatal stages, including the indexes of chao1, observed_species, PD_whole_tree and shannon. E0: control group, 2 weeks after birth; E1: EUGR group, 2 weeks after birth; F0: control group, 4 weeks after birth; F1: EUGR group, 4 weeks after birth

Both the species richness (observed_species) and Shannon index (shannon) showed a stable pattern of the curve, which indicated that the sequencing depth was sufficient to reflect the vast majority of the microbial diversity in the samples, even when the sample size was increased, limited new species were produced. During the 2nd week after birth, an obvious difference in the Shannon index appeared between theEUGR and control groups, but this difference was statistically insignificant. Meanwhile, there was no significant difference in any of the other indexes such as chao1, observed_species and PD_whole_tree between the two groups at 2 weeks or 4 weeks after birth (Fig. 3).

### Beta diversity analysis

**Fig. 4:**
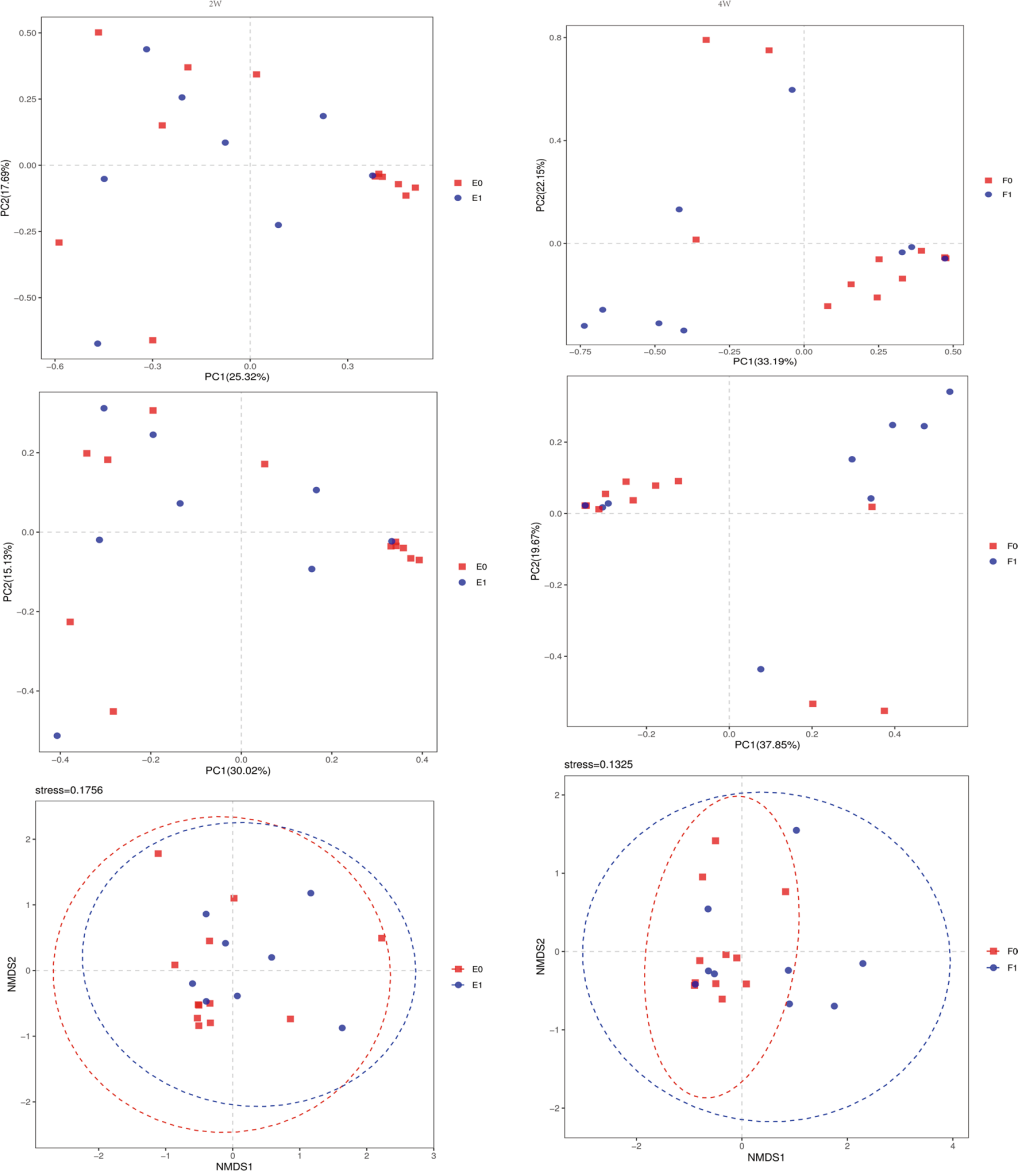
PCA, PCOA and NMDS analysis in the 2nd and 4th weeks after birth. The value of coordinate axis PC1/2 is the explanation rate of the overall difference. The midpoint represents the sample and the color represents the grouping. E0: control group, 2 weeks after birth; E1: EUGR group, 2 weeks after birth; F0: control group, 4 weeks after birth; F1: EUGR group, 4 weeks after birth

The results of the principal component analysis (PCA), principal co-ordinates analysis (PCOA) and non-metric multidimensional scaling (NMDS) analysis based on the OTU abundance in the stool samples showed that at 2 or 4 weeks after birth, the principal components of the intestinal microflora of the two groups could be roughly separated, despite their similarity. We use another dimensional reduction technique t-SNE and UMAP to interpret b diversity (see Additional file 1). This finding suggested a certain difference in the structure of intestinal microflora between the two groups (Fig. 4).

### LEfSe analysis

**Fig. 5:**
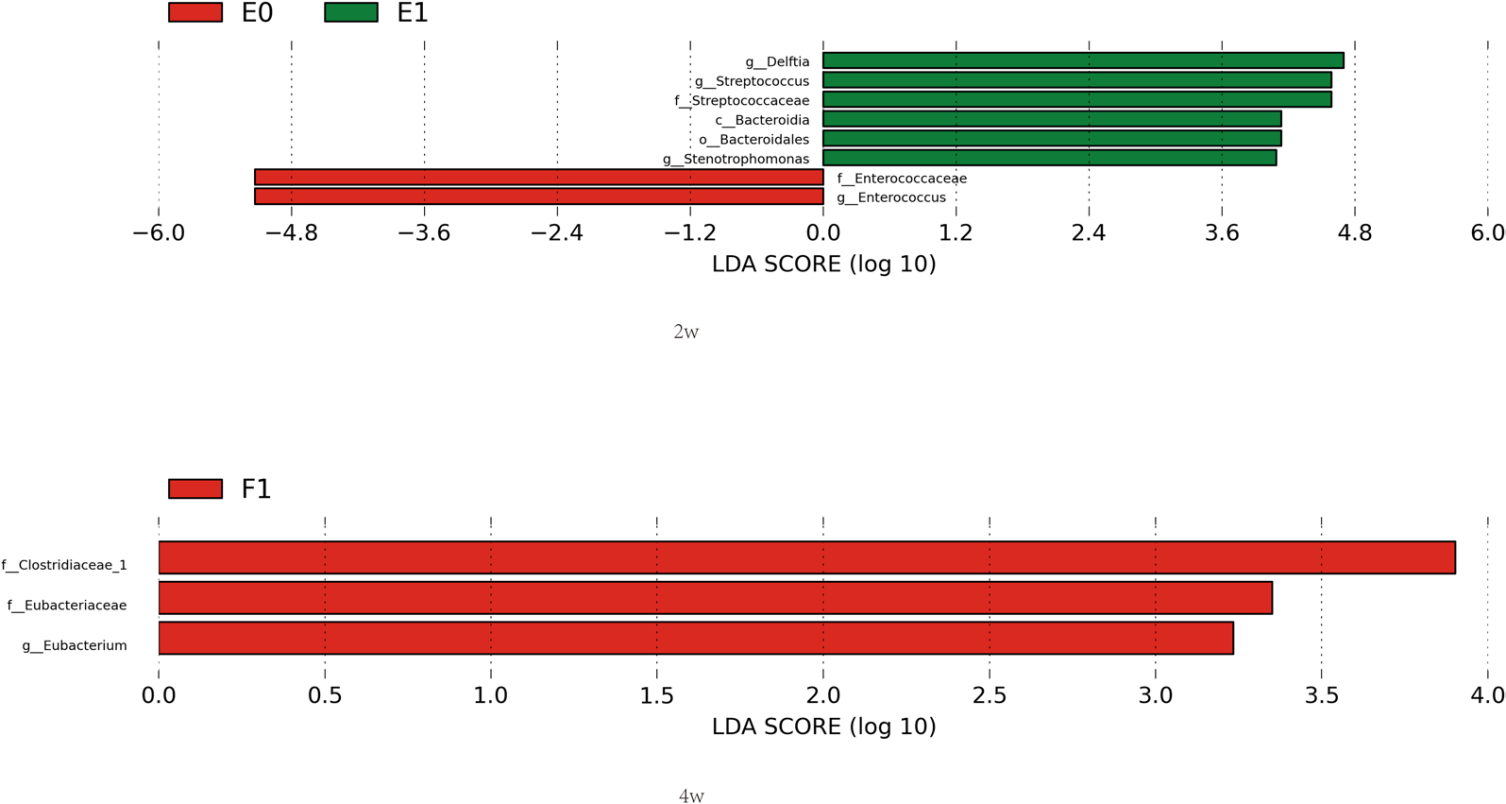
LEfSe analysis of the two groups at different postnatal stages. E0: control group, 2 weeks after birth; E1: EUGR group, 2 weeks after birth; F1: EUGR group, 4 weeks after birth

The linear discriminant analysis (LDA) effect size (LEfSe) can be used to compare multiple groups and perform subgroup comparison analysis, so as to find the species with an abundance that is significantly different between the groups. Based on the obtained species, an inter-group difference analysis was performed, followed by LDA to estimate the effect of each species on the difference. During the 2nd week after birth, the items with an absolute LDA higher than 4 in the EUGR group included Streptococcaceae, Streptococcus, Bacteroidetes, Bacteroidales and Stenotrophomonas, while those in the control group included Enterococcaceae and Enterococcus. On the other hand, during the 4th week after birth, the items with an absolute LDA higher than 3 in the EUGR group included Clostriaceae, Eubacteriaceae and Eubacterium (Fig. 5). Compared with 2 weeks after birth, there was no significant difference in the bacteria with an LDA score greater than 4.

### Comparison of the community composition

At 2 weeks after birth, there was a significant difference between the community principal components of the infants in the EUGR group and those in the normal control group on the family and genus levels. On the family level, the principal components in the control group were Enterococcaceae (53.95%) and Enterobacteriaceae (24.96%), while the Enterococcaceae content in the EUGR group significantly decreased to 23.45%, and there was no significant difference in the Enterobacteriaceae content (24.05%). Streptococcaceae accounted for 11.33% in the EUGR group and 2.58% in the control group, with a significant difference. On the genus level, the principal component of the control group was Enterococcus (53.92%), which accounted only for 26.44% in the EUGR group. The proportion of Streptococcus significantly differed between the two groups, accounting for 2.4% in the control group and 12.633% in the EUGR group (Additional file 2: Table S1). At 4 weeks after birth, there was no significant difference between the EUGR and control groups, and no Bifidobacterium was found in either group (Additional file 2: Table S2).

## Discussion

In the neonatal period, the intestinal microflora undergoes special dynamic changes, and its colonization and composition are affected by the body weight, gestational age, delivery mode, feeding mode as well as the living environment and drug use (such as antibiotics) [8, 9]. In this study, a total of 22 very preterm infants were included, among which 10 had growth retardation at 4 weeks after birth (10/22), and the incidence of EUGR was about 36–45%. In Tokyo area of Japan, the incidence of EUGR was reported to be 8.4% [10]. In 2016, Griffin et al. [11] analyzed the data of the California Perinatal Quality Care Collaborative from 2005 to 2012 and found that the incidence rate of EUGR at discharge of very low birth weight (VLBW) infants was 52.7 and 44.4% in the 1000–1249 g and 1250–1500 g weight groups, respectively. In 2017, Park et al. [12] reported that at 40 weeks of corrected gestational age, the incidence of EUGR was 58.4%. In China, the incidence of EUGR was higher. A multicenter survey of 572 VLBW infants in 15 hospitals across the country in 2015 showed that the incidence of EUGR at discharge was 80.9%, and the cases with a weight  <  3rd percentile accounted for 63.6%. The high incidence of EUGR reveals the challenging nature of the nutritional status of VLBW infants in China during hospitalization [13]. The lower the gestational age, the lower the birth weight and the higher the incidence of EUGR [14]. In our study, the incidence of EUGR was shown to be similar to that of foreign countries, which might be due to better management of preterm infant in the NICU. All the included very preterm infants were admitted to the NICU immediately after birth, and the intestinal microflora composition was detected by performing 16S rRNA high-throughput sequencing. The results showed a dynamic change in the diversity of intestinal flora of very preterm infants over time. The OTU number of the infants in the control group was significantly higher than that in the EUGR group, especially at 2 weeks after birth. The intestinal microflora in the EUGR group had a decreased diversity, but the difference compared with the control group was significantly reduced in the 4th week. Very preterm infants are faced with great challenges after birth, and their intestinal environment is very immature at first. Besides, the invasive operation of the NICU in the early stage of birth and the particularity of the NICU environment, which includes a more strict disinfection and sterilization system and a massive use of broad-spectrum antibiotics, result in a special composition of environmental microorganisms. Therefore, very preterm infants in the NICU have particular colonization model of intestinal bacteria, species types and diversity [15] (Additional file 2: Table S3–S4).

We performed a comparison between the composition of microbial community in the EUGR and control groups and found that Enterococcus was the dominant intestinal microflora in the two groups. However, the proportion of Enterococcus in the EUGR group was significantly lower than that in the control group, and the proportion of pathogenic bacteria, such as Streptococcus, was significantly increased. The LEfSe analysis showed that in the 2nd week after birth, Streptococcaceae and Streptococcus in the EUGR group and Enterococcaceae and Enterococcus in the control group were the most significantly different bacterial species between the two groups. Although the contents of Bacteroidetes, Bacteroidales and *Stenotrophomonas maltophilia* were also significantly different between the two groups, they had low proportions in the community composition of the two groups, which has no clear clinical significance. Streptococcus is a common pathogen that causes early-onset neonatal infection. Group B streptococcus (GBS) is the most common cause of early septicemia and meningitis in neonates. The mortality of early-onset GBS infection in full-term infants is 2–3%, while it reaches 20% in preterm infants and 30% in preterm infants with a gestational age of less than 33 weeks [16]. An analysis of 1,04,186 very preterm infants admitted to 312 NICUs in the United States from 1997 to 2011 showed that the rates of early-onset and late-onset GBS infection were 10.2 and 11.8%, respectively, such that early-onset infection increased the risk of death [17]. In China, the conducted studies showed that the incidence of neonatal infection in GBS-positive pregnant women was 29.8%, which was significantly higher than that in GBS-negative pregnant women (13.2%). Our results indicated that the proportion of Streptococcus significantly increased in the EUGR group at 2 weeks after birth, which suggests that growth restriction is more related to the disease status. Schwiertz et al. [18] analyzed the stool microbial diversity of 29 preterm infants at the NICU and 15 full-term infants within 4 weeks after birth and found that it took preterm infants 10 days to reach homeostasis. Jacquot et al. [19] showed that almost no Bifidobacterium was detected in preterm infants within 8 weeks after birth. Our results also indicated that intestinal microflora was close to steady state at 4 weeks after birth, but no Bifidobacterium was found in either group, which may be related to the special environment of the NICU.

EUGR represents a risk factor for neurodevelopmental abnormalities [20, 21]. Since the intestinal microflora maintains a bidirectional interaction with the central nervous system (CNS) through the gut-brain axis, its effect on newborns is not limited to the intestinal tract. Metabolites from the intestinal microflora disorders can destroy the blood–brain barrier (BBB), producing harmful components, which can then enter the brain more easily causing brain damage [22]. The intestinal colonization of Bifidobacterium can weaken the hypothalamic–pituitary–adrenal (HPA) axis response, and this inhibitory effect occurs in the early stage of life, which indicates that the original microbial exposure is necessary to inhibit the neural regulation of the HPA axis [23]. A recent study conducted by Bercik et al. [24] showed that after transferring the feces of the donor mice to the recipient mice, the recipient mice showed a similar behavioral phenotype to the donor mice, suggesting that the intestinal microbes can communicate with the brain through certain mechanisms.

## Conclusions

The establishment of intestinal microecology in very preterm infants undergoes dynamic changes over time and differs between preterm infants with EUGR and those with normal growth. The diversity and richness of the intestinal microflora in preterm infants in the NICU are significantly insufficient. Early detection of the intestinal microflora and early intervention can improve the growth of very preterm infants. Probiotics have a high strain specificity in the intestinal tract, and different probiotics have different functions; questions such as the colonization rate and time (short-term or long-term), suitable strains and doses remain to be investigated. In future work, we will investigate the types of probiotics that can effectively reduce the growth retardation of very preterm infants when they are early administrated.

## Supplementary Information

**Additional file1: Figure S1.** t-SNE and UMAP.

**Additional file 2: Table S1.** Comparison of communities at different levels at 2 weeks after birth (over 9%). **Table S2.** Comparison of communities at different levels at 4 weeks after birth (over 9%). **Table S3.** OTUs of the two groups at two weeks after birth. **Table S4.** OTUs of the two groups at four weeks after birth.

## Data Availability

Supporting data is available.

## Abbreviations

NICU: Neonatal intensive care unit
EUGR: Extrauterine growth retardation
WHO: World Health Organization
OUT: Operational taxonomic unit
PCA: Principal component analysis
NMDS: Non-metric multidimensional scaling
LefSe: Linear discriminant analysis (LDA) effect size
VLBW infants: Very low birth weight infants
GBS: Group B streptococcus
CNS: Central nervous system
BBB: Blood–brain barrier
HPA: Hypothalamic–pituitary–adrenal
