# Supplementary figures and images for: Effect of intestinal microecology on postnatal weight gain in very preterm infants in intensive care units
Source: Gut Pathog. 2021 Aug 2;13:49. doi: 10.1186/s13099-021-00445-1 (PMC8327448; doi:10.1186/s13099-021-00445-1)

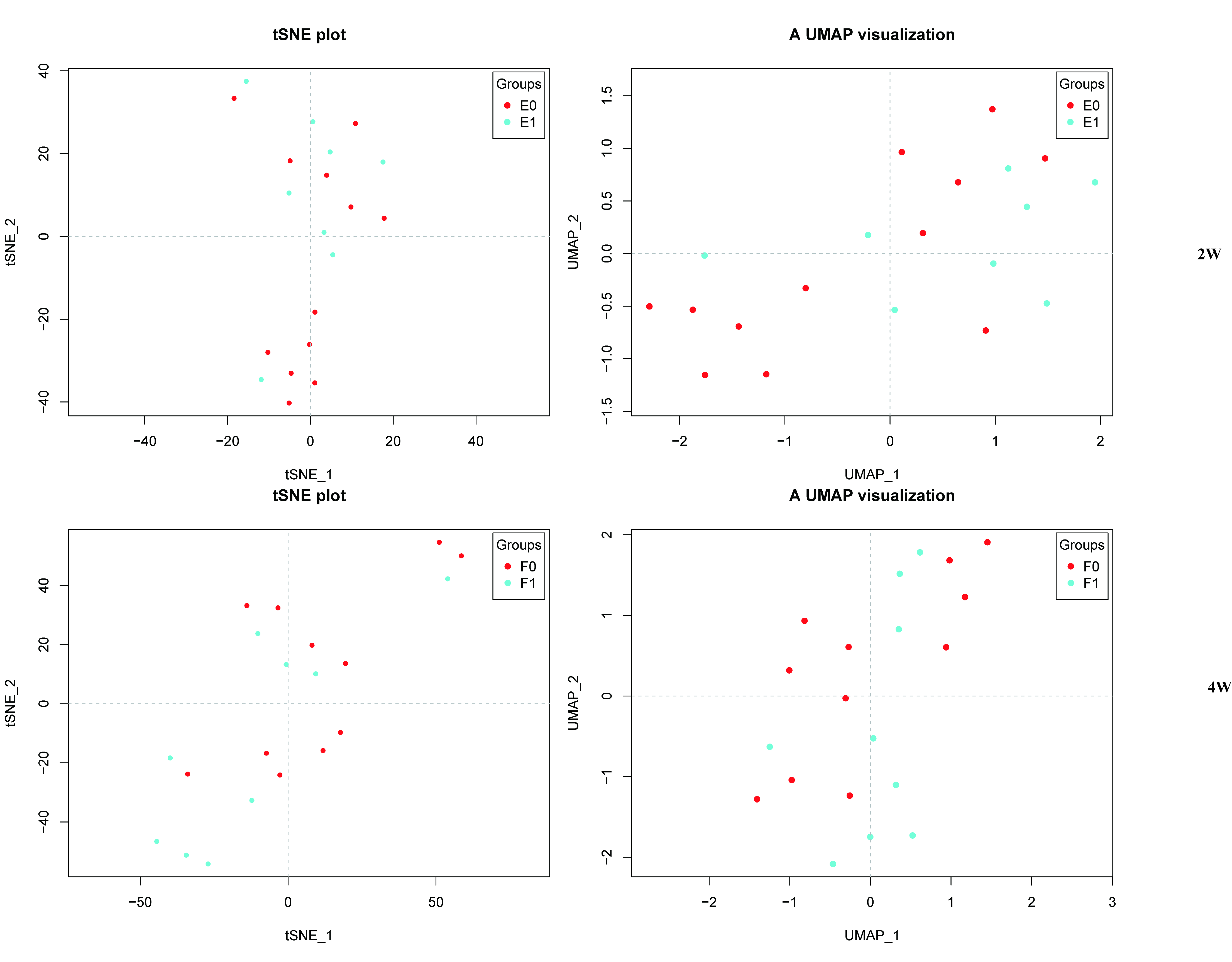

Supplement: Supplementary file 1 — Additional file1: Figure S1. t-SNE and UMAP. [file 13099_2021_445_MOESM1_ESM.tif]
