# Supplementary material for: Effect of intestinal microecology on postnatal weight gain in very preterm infants in intensive care units
Source: Gut Pathog. 2021 Aug 2;13:49. doi: 10.1186/s13099-021-00445-1 (PMC8327448; doi:10.1186/s13099-021-00445-1)
Supplement: Supplementary file 2 — Additional file 2: Table S1. Comparison of communities at different levels at 2 weeks after birth (over 9%). Table S2. Comparison of communities at different levels at 4 weeks after birth (over 9%). Table S3. OTUs of the two groups at two weeks after birth. Table S4. OTUs of the two groups at four weeks after birth. [file 13099_2021_445_MOESM2_ESM.doc]

Additional file 2: Table S1. Comparison of communities at different levels at 2 weeks after birth (over 9%)

|  |  | AGA | EUGR | P |
| --- | --- | --- | --- | --- |
| Phylum | p_Firmicutes | 68.49% | 56.37% | 0.48 |
|  | p_Proteobacteria | 31.18% | 29.3% | 0.75 |
|  | p_Actinobacteria | 1.89% | 9.6% | 0.23 |
| Class | c_Bacilli | 56.24% | 50.03% | 0.64 |
|  | c_Gammaproteobacteria | 24.11% | 26.69% | 0.53 |
|  | c_Clostridia | 6.06% | 5.78% | 0.46 |
|  | c_Actinobacteria | 1.61% | 9.16% | 0.24 |
| Order | o _Lactobacillales | 52.49% | 40.05% | 0.58 |
|  | o_Enterobacteriales | 23.59% | 24.05% | 0.53 |
|  | o_Bacillales | 3.74% | 9.93% | 0.16 |
| Family | f_Enterococcaceae | 49.54% | 26.47% | 0.18 |
|  | f_Enterobacteriaceae | 23.59% | 24.05% | 0.53 |
|  | f_Streptococcaceae | 2.4% | 12.63% | 0.01 |
|  | f_Staphylococcaceae | 3.63% | 9.43% | 0.21 |
| Genus | g_Enterococcus | 49.53% | 26.44% | 0.18 |
|  | g_Escherichia-Shigella | 10.71% | 2.11% | 0.26 |
|  | g_Streptococcus | 2.4% | 12.63% | 0.01 |
|  | g_Staphylococcus | 3.62% | 9.43% | 0.21 |
|  | g_Citrobacter | 4.45% | 9.16% | 0.11 |

Table S2. Comparison of communities at different levels at 4 weeks after birth (over 9%)

|  |  | AGA | EUGR | P |
| --- | --- | --- | --- | --- |
| Phylum | p_Firmicutes | 73.60% | 49.09% | 0.15 |
|  | p_Proteobacteria | 22.21% | 47.71% | 0.15 |
| Class | c_Bacilli | 71.54% | 46.53% | 0.11 |
|  | c_Gammaproteobacteria | 21.19% | 46.66% | 0.18 |
| Order | o _Lactobacillales | 69.83% | 43.08% | 0.15 |
|  | o_Enterobacteriales | 20.90% | 41.21% | 0.46 |
| Family | f__Enterococcaceae | 60.71% | 36.92% | 0.21 |
|  | f__Enterobacteriaceae | 20.90% | 41.21% | 0.46 |
| Genus | g_Enterococcus | 60.71% | 36.89% | 0.21 |
|  | g_Escherichia-Shigella | 4.78% | 8.55% | 0.26 |
|  | g_Citrobacter | 14.83% | 5.72% | 0.30 |
|  | g_Klebsiella | 0.88% | 14.51% | 0.36 |

Table S3. OTUs of the two groups at two weeks after birth

| ID | mean_E0 | mean_E1 | p_value |  |
| --- | --- | --- | --- | --- |
| OTU_231 | 0 | 3.13E-05 | 0.007601641 | k_Bacteria; p_Proteobacteria; c_Gammaproteobacteria; o_Pasteurellales; f_Pasteurellaceae; |
| OTU_1100 | 0 | 6.25E-05 | 0.007887578 | k_Bacteria; p_Nitrospirae; c_Nitrospira; o_Nitrospirales; f_Nitrospiraceae; |
| OTU_1163 | 0 | 4.69E-05 | 0.007984101 | k_Bacteria; p_Actinobacteria; c_Coriobacteriia; o_Coriobacteriales; f_Coriobacteriaceae; |
| OTU_283 | 0.000151 | 0.00122 | 0.020885052 | k_Bacteria; p_Proteobacteria; c_Betaproteobacteria; o_Burkholderiales; f_Alcaligenaceae; |
| OTU_654 | 0 | 2.35E-05 | 0.024920361 | k_Bacteria; p_Proteobacteria; c_Deltaproteobacteria; o_Myxococcales; f_Haliangiaceae; |
| OTU_873 | 0 | 0.000109 | 0.025347319 | k_Bacteria; p_Bacteroidetes; c_Sphingobacteriia; o_Sphingobacteriales; f_Chitinophagaceae; |
| OTU_105 | 0.000198 | 0.002369 | 0.025628984 | k_Bacteria; p_Gemmatimonadetes; c_Longimicrobia; o_Longimicrobiales; f_Longimicrobiaceae; |
| OTU_818 | 3.65E-05 | 0.000149 | 0.034094824 | k_Bacteria; p_Bacteroidetes; c_Bacteroidia; o_Bacteroidales;  f_Bacteroidales S24-7_group; |
| OTU_102 | 0.000396 | 0.019022 | 0.038194222 | k_Bacteria; p_Proteobacteria; c_Deltaproteobacteria; o_Desulfurellales; f_Desulfurellaceae; |
| OTU_480 | 0.000109 | 0.000321 | 0.039438926 | k_Bacteria; p_Actinobacteria; c_Thermoleophilia; o_Solirubrobacterales; f_FFCH13075; |
| OTU_72 | 3.13E-05 | 0 | 0.041226833 | k_Bacteria; p_Gemmatimonadetes; c_S0134_terrestrial_group; o_unidentified; f_unidentified; |
| OTU_41 | 0.021428 | 0.113906 | 0.044862271 | k_Bacteria; p_Gemmatimonadetes; c_Longimicrobia; o_Longimicrobiales; f_Longimicrobiaceae; |
| OTU_876 | 1.04E-05 | 5.47E-05 | 0.047724028 | k_Bacteria; p_Bacteroidetes; c_Sphingobacteriia; o_Sphingobacteriales; f_Chitinophagaceae; |

Table S4. OTUs of the two groups at four weeks after birth

| ID | mean_F0 | mean_F1 | p_value |  |
| --- | --- | --- | --- | --- |
| OTU_1040 | 6.19E-06 | 0.000447 | 0.006411 | k_Bacteria; p_Gemmatimonadetes; c_Gemmatimonadetes; o_Gemmatimonadales; f_Gemmatimonadaceae; |
| OTU_1027 | 1.24E-05 | 9.84E-05 | 0.009506 | k_Bacteria; p_Gemmatimonadetes; c_Gemmatimonadetes; o_Gemmatimonadales; f_Gemmatimonadaceae; |
| OTU_616 | 0 | 4.54E-05 | 0.016641 | k_Bacteria; p_Firmicutes; c_Erysipelotrichia; o_Erysipelotrichales; f_Erysipelotrichaceae; |
| OTU_1036 | 1.24E-05 | 0.000151 | 0.019399 | k_Bacteria; p_Gemmatimonadetes; c_Gemmatimonadetes; o_Gemmatimonadales; f_Gemmatimonadaceae |
| OTU_1241 | 6.19E-05 | 0 | 0.023739 | k_Bacteria;p_Actinobacteria |
| OTU_40 | 0 | 2.27E-05 | 0.042933 | k_Bacteria; p_Firmicutes; c_Bacilli; o_Bacillales; f_Bacillaceae; |
| OTU_351 | 0 | 0.000114 | 0.043546 | k_Bacteria; p_Firmicutes; c_Negativicutes; o_Selenomonadales; f_Veillonellaceae; |
| OTU_892 | 0 | 5.30E-05 | 0.043546 | k_Bacteria; p_Bacteroidetes c_Sphingobacteriia; o_Sphingobacteriales; f_Sphingobacteriaceae; |
| OTU_370 | 0 | 4.54E-05 | 0.043546 | k_Bacteria; p_Proteobacteria; c_Betaproteobacteria; o_Burkholderiales; f_Comamonadaceae; |
| OTU_868 | 0 | 3.79E-05 | 0.043546 | k_Bacteria; p_Bacteroidetes; c_Flavobacteriia; o_Flavobacteriales; f_Flavobacteriaceae; |
| OTU_1039 | 0 | 3.79E-05 | 0.043546 | k_Bacteria; p_Gemmatimonadetes; c_Gemmatimonadetes; o_Gemmatimonadales; f_Gemmatimonadaceae; |
| OTU_41 | 0 | 3.03E-05 | 0.043546 | k_Bacteria; p_Gemmatimonadetes; c_Longimicrobia; o_Longimicrobiales; f_Longimicrobiaceae; |
| OTU_446 | 0 | 3.03E-05 | 0.043546 | k_Bacteria; p_Actinobacteria; c_Thermoleophilia; o_Solirubrobacterales; f_Gsoil-1167; |
| OTU_123 | 0 | 0.000734 | 0.043751 | k_Bacteria; p_Firmicutes; c_Bacilli; o_Bacillales |
